# The Dynamics of the Ferret Immune Response During H7N9 Influenza Virus Infection

**DOI:** 10.3389/fimmu.2020.559113

**Published:** 2020-09-24

**Authors:** William S. J. Horman, Thi H. O. Nguyen, Katherine Kedzierska, Jeffrey Butler, Songhua Shan, Rachel Layton, John Bingham, Jean Payne, Andrew G. D. Bean, Daniel S. Layton

**Affiliations:** ^1^Department of Microbiology and Immunology, The Peter Doherty Institute for Infection and Immunity, University of Melbourne, Parkville, VIC, Australia; ^2^Commonwealth Scientific and Industrial Research Organisation Health and Biosecurity, Australian Centre for Disease Prevention, East Geelong, VIC, Australia; ^3^Commonwealth Scientific and Industrial Research Organisation, Australian Centre for Disease Prevention, East Geelong, VIC, Australia

**Keywords:** influenza, H7N9, ferrets, antigen presenting cells, animal model, zoonoses

## Abstract

As the recent outbreak of SARS-CoV-2 has highlighted, the threat of a pandemic event from zoonotic viruses, such as the deadly influenza A/H7N9 virus subtype, continues to be a major global health concern. H7N9 virus strains appear to exhibit greater disease severity in mammalian hosts compared to natural avian hosts, though the exact mechanisms underlying this are somewhat unclear. Knowledge of the H7N9 host-pathogen interactions have mainly been constrained to natural sporadic human infections. To elucidate the cellular immune mechanisms associated with disease severity and progression, we used a ferret model to closely resemble disease outcomes in humans following influenza virus infection. Intriguingly, we observed variable disease outcomes when ferrets were inoculated with the A/Anhui/1/2013 (H7N9) strain. We observed relatively reduced antigen-presenting cell activation in lymphoid tissues which may be correlative with increased disease severity. Additionally, depletions in CD8^+^ T cells were not apparent in sick animals. This study provides further insight into the ways that lymphocytes maturate and traffic in response to H7N9 infection in the ferret model.

## Introduction

In recent years cases of zoonotic strains of avian influenza (AI) causing severe disease in humans have caused significant global concern, with fears that these viruses may lead to devastating pandemic events in future (1, 2). One such group of viruses are strains of H7N9 influenza virus, which have caused over 1,500 cases of infection (with a ~40% mortality rate) in humans since their first detection in 2013 (3, 4). Of most concern with the H7N9 viruses has been the ability for this virus to present as a low pathogenicity virus in its native avian hosts and yet present with severe clinical symptoms and death in humans, without obtaining virulence factors such as multi-basic cleavage sites usually required for high pathogenicity infections. Deciphering the immune cellular mechanisms associated with disease severity progression will provide a better understanding as to why H7N9 infections can produce severe disease in humans.

Changes in leukocyte subsets have previously been shown to correlate with more severe outcomes during AI infection, with decreases in T cell populations commonly reported in both human (H7N9 virus) and avian (H5N6 virus) hosts (5, 6). Decreases in T lymphocytes are often accompanied by upregulation of several pro-inflammatory cytokines such as interferons (IFN), most notably IFN-γ, as well as interleukins (IL) such as IL-6 (7). Overproduction of cytokines or hypercytokinemia has also been identified as a key contributing factor in severe AI pathogenesis in both chickens and macaques, where induction of pro-inflammatory cytokines is associated with cellular apoptosis and tissue damage (8, 9). Furthermore, macrophages have been shown to be predominantly pro-inflammatory responders to H7N9 strains in a mouse model (10), though highly pathogenic strains such as H5N1 and H7N9 display attenuated macrophage inflammation responses compared to seasonal strains such as H1N1 and H3N2 (11, 12). While hypercytokinemia is common amongst many AI virus infections, H7N9 strains such as the human infecting A/Anhui/1/2013 virus have been associated with dampened IFN responses in humans (13, 14). Furthermore, they have previously been associated with an attenuated humoral immune response in the mouse model (15). These studies demonstrate the unpredictable nature and wide spectrum of pathogenicity of AI viruses.

Investigations into the pathogenesis and transmission of many human-infecting influenza viruses have been conducted in ferrets, as the clinical presentation in these animals is considered a more robust representation of human illness when compared to mice (16). However, only a limited number of studies exist looking at influenza-specific immunity in ferrets, which have primarily focused on seasonal influenza strains (17–19). In this study, we aimed to examine the ferret immune response to H7N9 influenza virus infection by analyzing leukocyte population variation associated with disease pathogenesis. This study found the ferret model may allow for increased knowledge of the outcomes of H7N9 infections and help in boosting our understanding of both this model and of these viruses in readiness for potential future outbreaks.

## Methods

### Ethics

All procedures described here were reviewed and approved by the Commonwealth Scientific and Industrial Research Organization (CSIRO), Australian Center for Disease Prevention (ACDP) Animal Ethics Committee (AEC#1861) and were performed in accordance with the Australian Code for the Care and Use of Animals for Scientific Research (8th Edition 2013).

### Virus

Influenza virus A/Anhui/1/2013(H7N9) used in this study was propagated by allantoic cavity inoculation of 9–11-days of embryogenesis specific-pathogen-free (SPF) embryonated chicken eggs. The virus stock was titrated in chicken eggs and the 50% egg infectious dose (EID_50_) /mL was calculated according to Reed and Muench (20). All *in vitro* and *in vivo* work involving live virus was conducted within biosafety level three facilities at ACDP. Animal work was performed using personal protective equipment and powered air purifying respirators.

### Ferret Viral Challenge Studies

Fitch ferrets that were ~6 months of age (CSIRO Werribee Animal Facility) and serologically negative by hemagglutination inhibition (HI) assay for H7N9 were used in this study. Prior to initiation of the study, all ferrets were free from signs of clinical disease. Body temperatures were measured using an implantable subcutaneous microchip (Destron Fearing, Delray Beach, FL, USA). Baseline body weights and temperatures were obtained for 3 consecutive days before challenge (i.e., day −3, −2, and −1) and on the day just prior to the challenge (day 0).

Six ferrets were infected intranasally with 1 × 10^6^ EID_50_ of virus diluted in 0.5 mL of sterile PBS, and four ferrets were mock-infected with an equivalent dilution of allantoic fluid collected from SPF chicken eggs in sterile PBS as non-infected controls. Following viral challenge, ferrets were monitored daily for body weight, temperature, and clinical signs of illness (including sneezing, lethargy, nasal discharge, diarrhea, and neurological dysfunction) for the duration of the study. Blood samples were collected every second day. Animals were anesthetized with ketamine/xylazine, and blood samples of 200–250 μL were taken from the jugular or axillary vein on days 1, 3, 5, and from the heart at the time of euthanasia for the terminal bleed. For virus titration, nasal washes were collected on days 1, 3, 5, and upon euthanasia, as described previously (6, 21).

All ferrets were euthanised on day 7 (study endpoint) or earlier due to ethical endpoints (≥10% weight loss or escalation of clinical signs).

### Histopathology

Histological analysis of ferret tissues following infection was performed as previously described (22). Tissues were fixed in 10% neutral-buffered formalin for at least 24 h, processed into paraffin wax, cut and stained using haematoxylin and eosin for examination for histopathological lesions. Consecutive tissue sections were stained in an immunohistochemistry (IHC) test for influenza A virus nucleoprotein (22).

### Sample Collection and Processing

Lung, spleen, and mediastinal lymph nodes were harvested and processed, as previously described (17). Briefly, lung samples were manually minced using a scalpel followed by enzymatic digestion (150 U/mL Collagenase I and 100 U/mL DNase I) while single-cell suspensions of spleen and lymph node samples were prepared by passing the tissue though a 70 μm strainer. Peripheral blood mononuclear cells were isolated by hypotonic lysis of red blood cells using erythrocyte lysing solution (0.15 M NH_4_Cl, 10 mM KHCO_3_, and 1mM EDTA pH 7.3). Viral titers in tissues were measured on MDCK cells by standard TCID_50_ assay.

### Quantitative Real-Time PCR (Qrt-Pcr)

Relative expression of ferret immune genes was assessed using a StepOnePlus™ Real-Time PCR System and the comparative threshold cycle (Ct) method according to manufacturer's instructions (Applied Biosystems, Foster City, CA, USA). Relative gene expression was calculated using mean values obtained from ΔΔCt relative to the housekeeper gene (GAPDH), with each ferret compared to the average of the control ferrets for each gene. Primers for ferret cytokines, as well as relative gene expression calculations, were obtained from Carolan et al. (23).

### Flow Cytometry

Cells processed from ferret tissues were stained with anti-CD4 (AlexaFluor 488, CSIRO ACDP sourced from WEHI (24), Geelong, VIC, Australia), anti-CD8 (PE, clone OKT8, eBioscience, CA, USA), anti-GL7 (AlexaFluor 647, clone GL7, BD Phamingen, San Diego, CA, USA), anti-MHC-II (biotin, clone CAT82A, Kingfisher, Saint Paul, MN, USA), and anti-CD11b (AlexaFluor 647, clone M1/70, BD Pharmingen, San Diego, CA, USA). Cells were stained for 1 h at 4°C, washed in FACS buffer (PBS, 4% FCS, 0.01% Sodium Azide) and analyzed using the LSR II (Becton-Dickinson, Franklin Lakes, NJ, USA). Flow cytometry data were analyzed using FlowLogic software (Version 7.2.1, Inivai Technologies, Mentone, VIC, Australia).

### Serology

To assess antibody responses, serum was collected prior to infection and at the point of euthanasia. Haemagglutinin Inhibition assays were performed on RDE treated sera by using homologous antigen (Influenza A/Anhui/1/2013) as per the standard method. HI titers were expressed as the reciprocal of the highest serum dilution causing complete inhibition of hemagglutination.

### Functional IFN-γ Assays

Ferret lung and spleen cells were pelleted by centrifugation at 1,500 × g_max_ for 5 min and washed in PBS. Cells (1.25 × 10^4^/96-well) were cultured with or without live H7N9 virus for 48 h at 37°C/5% CO_2_. The Ferret IFN-γ ELISA Development Kit (ALP) (Mabtech, Stockholm, Sweden) was used to determine the quantity of IFN- γ secreted by cells *ex vivo* at endpoint and after restimulation with live H7N9 virus (MOI 0.1). ELISA plates were measured using a Multiskan Ascent Plate Reader with Ascent Software Version 2.6 (ThermoFisher, Waltham, MA, USA). IFN-γ-producing cells were detected using a ferret IFN-γ ELISpot assay, as per the manufacturer's instructions (Mabtech, Stockholm, Sweden).

### Statistical Analysis

For analyzing the infected ferrets compared to the non-infected controls, all six ferrets were grouped regardless of timepoint to give an overall view of the infection time course. For these analyses, student *t*-tests were conducted between the two groups. Time course data was analyzed by 2-way ANOVA.

## Results

### Ferrets Can Exhibit Moderate to Severe Clinical Signs Following H7N9 Virus Infection

**Figure 1:**
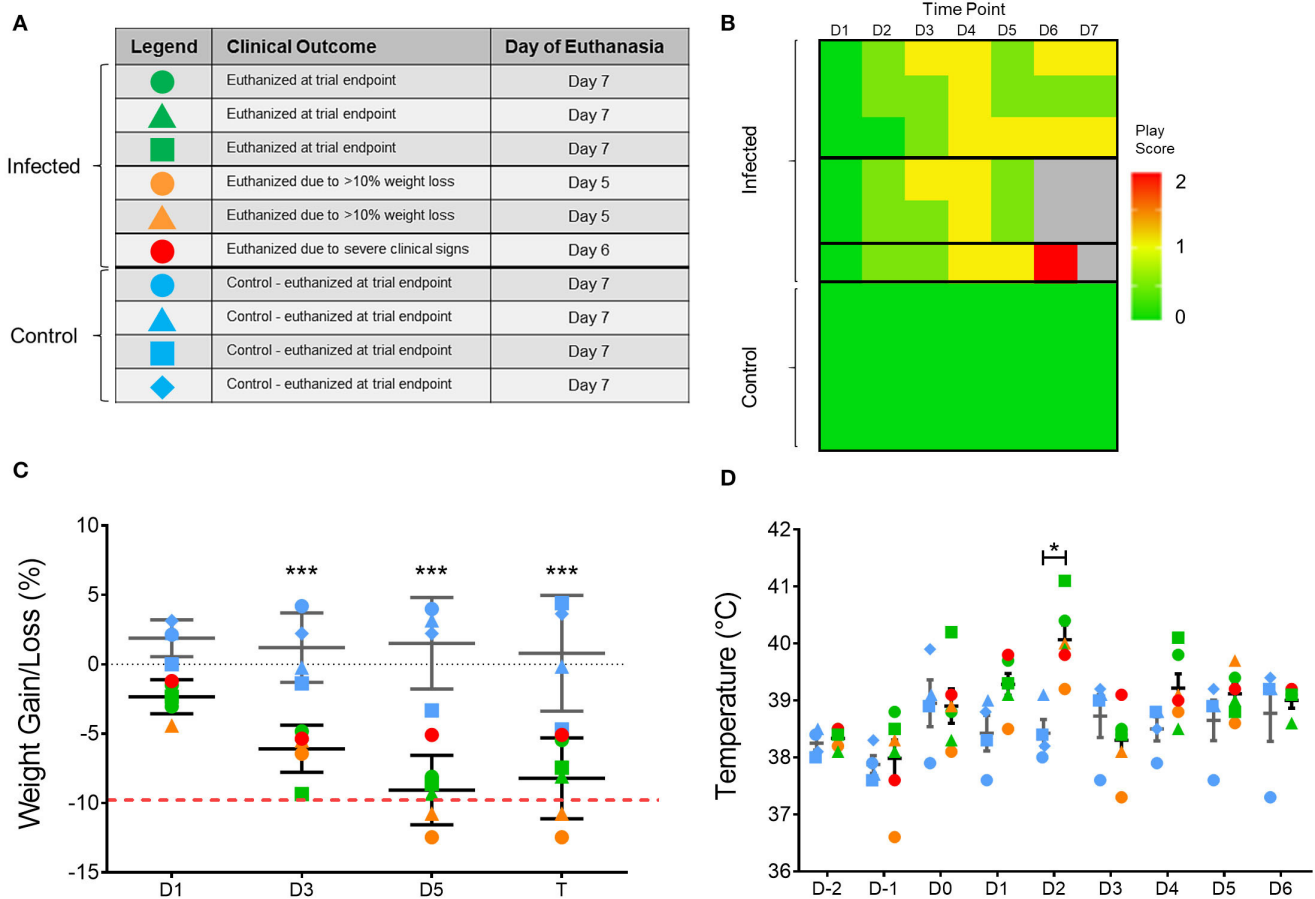
Variation in clinical outcome amongst study ferrets. **(A)** Summary of ferret characteristics and classification of ferrets according to clinical signs and time of euthanasia. Blue represents the uninfected controls; green represents ferrets which survived until trial endpoint (day 7); yellow represents ferrets with few clinical signs but reached ethical weight loss point (day 5); and red represents the ferret which showed severe clinical signs and was euthanised at (day 6). **(B)** Individual play scores measured for ferrets daily, with 0 signifying no signs, 1 minor clinical signs, and 2 moderate-to-severe clinical signs. Ferrets reaching a score of 2 were euthanised for ethical reasons. **(C)** Body weight and **(D)** temperature of each ferret throughout the study according to the legend shown in **(A)**. Mean and SD are shown, *n* = 6 for H7N9-infected ferrets, *n* = 4 for control ferrets. Weight data are plotted as a percentage change from the initial D-3 weight recording on days post-infection. Time points marked with (*) indicate significant differences between infected and controls where *p* < 0.05, and (***) indicates *p* < 0.001.

To assess the impact of H7N9 virus in ferrets, viral pathogenicity was firstly assessed based on observation of clinical signs of infected ferrets throughout the 7-day study (Figure 1). From the six H7N9-infected ferrets, three ferrets showed little to no clinical signs and survived until the study endpoint (Day 7). Of the remaining ferrets, two showed mild clinical signs but severe weight loss, leading to euthanasia at day 5 post-infection. One ferret, however, showed moderate to severe clinical signs consistent with those seen in highly pathogenic avian influenza infections (25), and was euthanised at day 6 due to escalation of these clinical signs (Figure 1A). This ferret's increase in clinical signs was identified by a play score of two, with play scores >1 signifying moderate-to-severe clinical signs (Figure 1B). Significant weight losses (>5% compared to baseline, *p* < 0.0001) were observed in all the infected ferrets when compared to the control ferrets from day 3 until the study endpoint (Figure 1C). Similarly, infected ferrets also showed a significant increase (*p* < 0.05) in body temperature at day 2 post-infection, with an average temperature of 40.1 ± 0.6°C compared to the controls at 38.4 ± 0.4°C (Figure 1D). Consequently, H7N9 infection in ferrets resulted in variable clinical symptoms, but overall body weight losses and heightened body temperatures at 48 h post-infection, which are within the typical timeframe for onset of influenza illness in humans.

### Lack of Viral Clearance and Lower Antibody Responses Were Associated With Worsened Disease Progression

**Figure 2:**
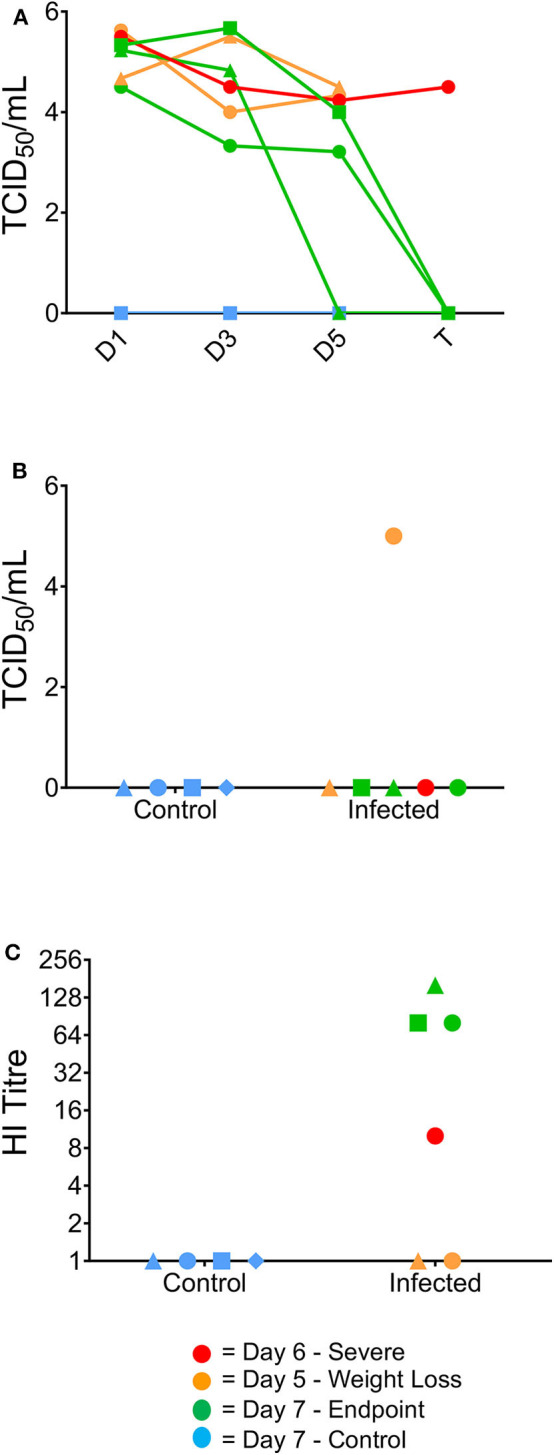
Virological and serological data correlating to clinical outcome. Virus titres (TCID_50_/ml) in nasal washes collected from the ferrets on days 1, 3, 5, and 7 post-infection **(A)**, and in the lung at the time of humane euthanasia **(B)**. Day 7 ferrets showed viral clearance by study endpoint, whilst earlier timepoint ferrets did not clear the virus by euthanasia. **(B)** One of the 2 day five ferrets was the only ferret to show live virus in the lungs post-study. **(C)** Haemagglutination inhibition assay titres in ferret serum samples collected from terminal bleeds. Day 7 ferrets also exhibited elevated serum titres by HI assay from terminally collected samples, whereas the 1 day six ferret was the only culled ferret to have a positive serum sample.

To further examine the clinical progression between the infected ferrets, viral titres from the nasal washes were determined to assess whether differences in viral replication or clearance were correlative with worsened disease progression. Titres >4.0 log_10_ TCID_50_/mL were obtained for all infected ferrets at day 1 post-infection and virus was still detected in all ferrets at day 3 post-infection (>3.0 log_10_ TCID_50_/mL, Figure 2A). One ferret had cleared the virus by day 5 post-infection, while the other two ferrets that survived until the end of the study showed viral clearance at day 7 (Figure 2A). Neither the day 5 nor the day 6 ferrets showed viral clearance by the point of euthanasia. Only one ferret euthanised on day 5 showed live virus in the lung (>4.0 log_10_/mL, Figure 2B). Additionally, antibody titres were measured in the ferret sera, with animals euthanized on day 7 showing the highest HI titres (all >64) and the ferret euthanized on day 6 showed a HI titer >8. The two ferrets euthanised at day 5 had no detectable HI titer (Figure 2C).

### Infected Ferrets Showed Variability in Pathological Outcomes in Respiratory Tissues

**Figure 3:**
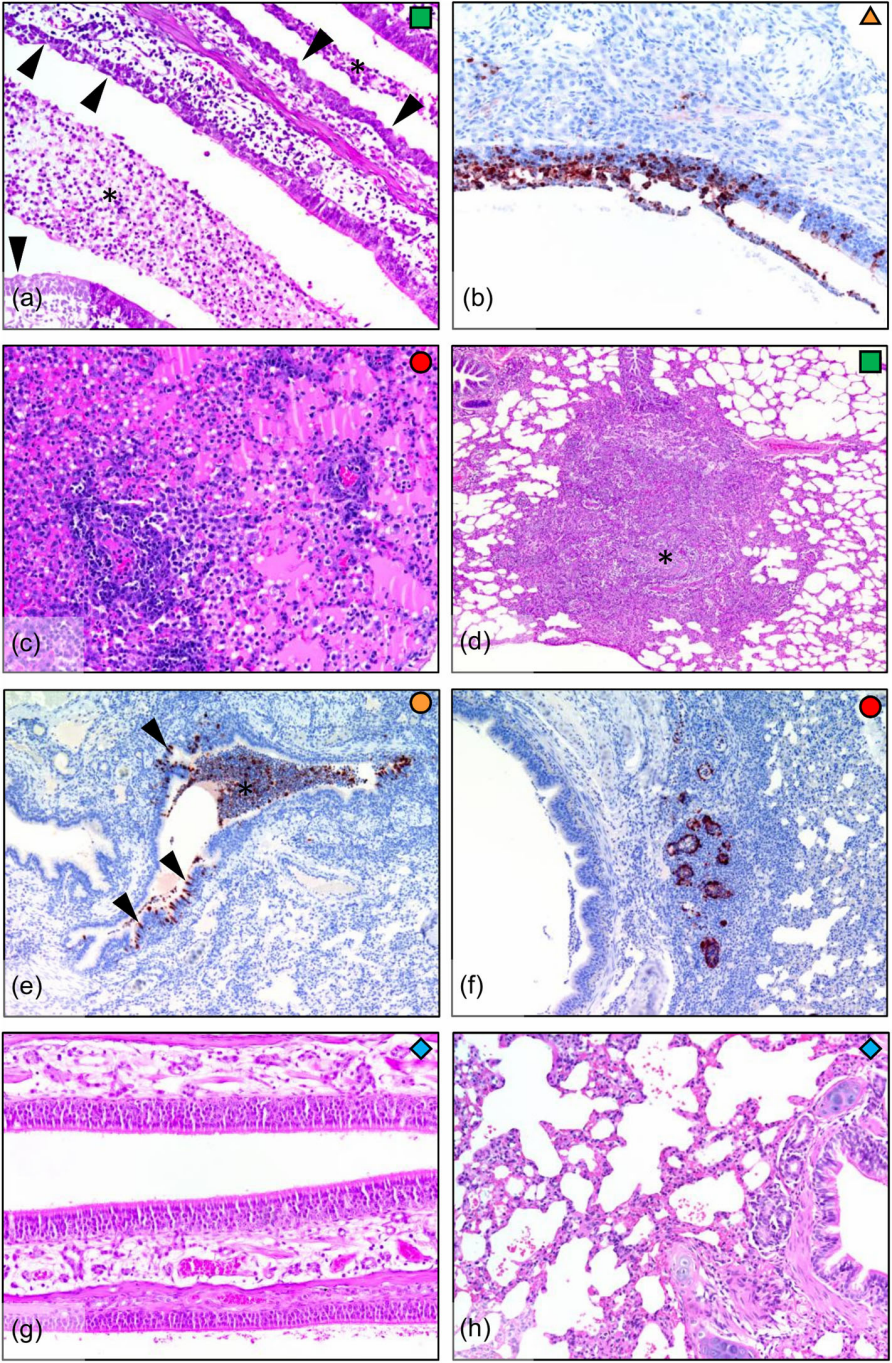
Histopathology in ferrets infected with H7N9 virus. **(a)** Infected nasal turbinate, showing epithelial metaplasia (arrowheads) and suppurative inflammatory exudate (*) in airways (day 7, HE stain). **(b)** Nasal turbinate during early infection (day 5) showing viral infection of turbinate epithelium (IHC stain). **(c)** Diffuse interstitial pneumonia, showing severe alveolar oedema and inflammatory cell infiltration around blood vessels and into the alveolar spaces (lung, day 6). **(d)** Broncho-interstitial pneumonia, showing a large focal lesion involving an obliterated airway and surrounding inflammation into the interstitium (lung, day 7). **(e)** Bronchitis, showing infection of bronchial epithelium (arrowheads) and filling of the airway with infected sloughed cells and inflammatory cells (*). In this example, the bronchial inflammatory response is minimal, indicating that the lesion is in the early stages (lung, day 5). **(f)** Bronchial adenitis, showing viral antigen and necrosis of bronchial glands (lung, day 6). **(g)** No pathology was observed in the nasal turbinates, or **(h)** in the lungs of the uninfected controls (day 7).

Infected ferrets showed a variety of pathological outcomes following viral challenge. Challenged ferrets exhibited epithelial metaplasia in the nasal turbinates (Figure 3a), with ferrets euthanised at the earlier day 5 time point exhibiting viral infection of the nasal epithelium (Figure 3b). The day 6 ferret showed the most severe lung pathology, with the lungs of this ferret showing diffuse interstitial pneumonia, with severe alveolar oedema and inflammatory cell infiltration in the alveolar spaces and around the blood vessels (Figure 3c). Other infected ferrets showed broncho-interstitial pneumonia and interstitial inflammation (Figure 3d) and bronchitis, with infected epithelial cells and early stage lesions observed in one of the day 5 ferrets (Figure 3e). Bronchial adenitis was also present in some of the infected ferrets, with necrosis of the bronchial glands observed along with viral antigen (Figure 3f). These changes in pathology contrast to what is seen in the healthy nasal turbinates (Figure 3g), and lung (Figure 3h) of uninfected ferrets where no lesions were present. The number of localized lesions and qualitative assessment of epithelial metaplasia was also recorded to support the histopathological findings (Supplementary Figure 1). Here, we observed epithelial metaplasia in the turbinates of all ferrets sampled, as well as several occurrences of bronchio-interstitial pneumonia and bronchio-adenitis. We also observed viral antigen in a number of tissues sampled (Supplementary Figure 1), though interestingly, viral antigen was not observed in the diffuse interstitial pneumonia of lung lesions associated with the ferret euthanized due to clinical disease.

### Disease Severity Is Associated With Different Expression Profiles of Pro-inflammatory Cytokines

**Figure 4:**
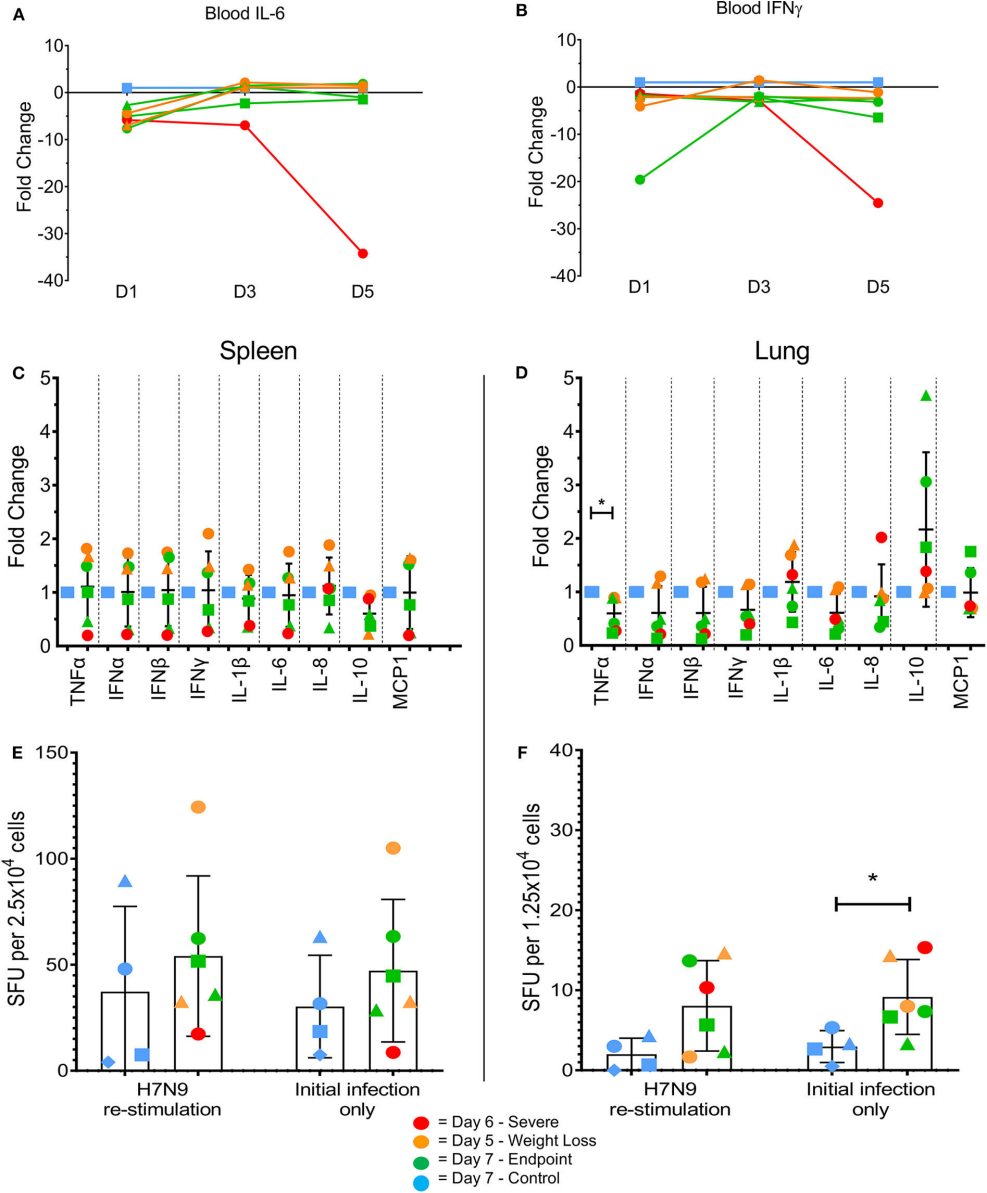
Changes in pro-inflammatory cytokine levels in multiple tissues by qPCR and ELISpot assay. Immune cytokine levels were determined using TaqMan qPCR assays on cDNA prepared from the RNA of lysed tissues. In the blood samples the day 5 ferret showed large decreases in **(A)** IL-6 and **(B)** IFN-γ at day 5 post-infection. **(C)** In the spleen, little variation was seen between control and infected samples. Levels of IFN-γ producing cells were determined by ELISpot assay. **(D)** In the lung, H7N9-infected ferrets showed a significant fold change in TNFα. **(E)** In the lung, H7N9-infected ferrets showed significantly greater levels (*p* = 0.0389) of spots compared to non-infected controls without re-stimulation with H7N9. **(F)** In the spleen, little variation was seen between control and infected samples regardless of re-stimulation. Data marked with (*) indicate *p* < 0.05.

Changes in certain pro-inflammatory cytokines (including IL-6, TNFα, and IFNγ) have been associated with H7N9 disease severity in humans and animal models (7). We measured levels of mRNA transcripts of several pro-inflammatory cytokines commonly associated with influenza infections. In the blood there were few differences between the groups, with MCP1 the only tested cytokine showing an average fold increase >5-fold compared to the controls at day 1 post-infection (data not shown). However, intriguingly the ferret euthanised on day 6 showed a large decrease in IL-6 (Figure 4A, 34-fold) and IFN-γ (Figure 4B, 25-fold) transcript levels on day 5. This also coincided with a lack of detectible IFN-γ in terminal serum by ELISA, though this trend was consistent across all tested ferrets (data not shown). In the spleen at endpoint, there appeared to be variable outcomes for the day 7 ferrets, with somewhat increased pro-inflammatory cytokine levels such as type I IFNs (IFN-α and IFN-β) in the day 5 ferrets, and decreased levels in the day 6 ferret compared to the controls (Figure 4C). In the lung, TNFα was significantly reduced (Figure 4D, *p* < 0.05) in the infected animals, though no other consistent trends were seen. The ability to produce IFN-γ *ex vivo* or after restimulation with live H7N9 virus was also assessed in spleen (Figure 4E) and lung cells (Figure 4F). There were no significant differences except for in the infected lungs, where IFN-γ-producing cells were significantly higher compared to the control ferrets *ex vivo* (*p* = 0.0389). Based on these findings, our ferret infection model showed observable changes in the cytokine profiles at a key site of infection in the lung compared to non-infected controls.

### T Cell Populations Increase in the Infected Lungs, but No Apparent Change Is Present in the Peripheral Blood

**Figure 5:**
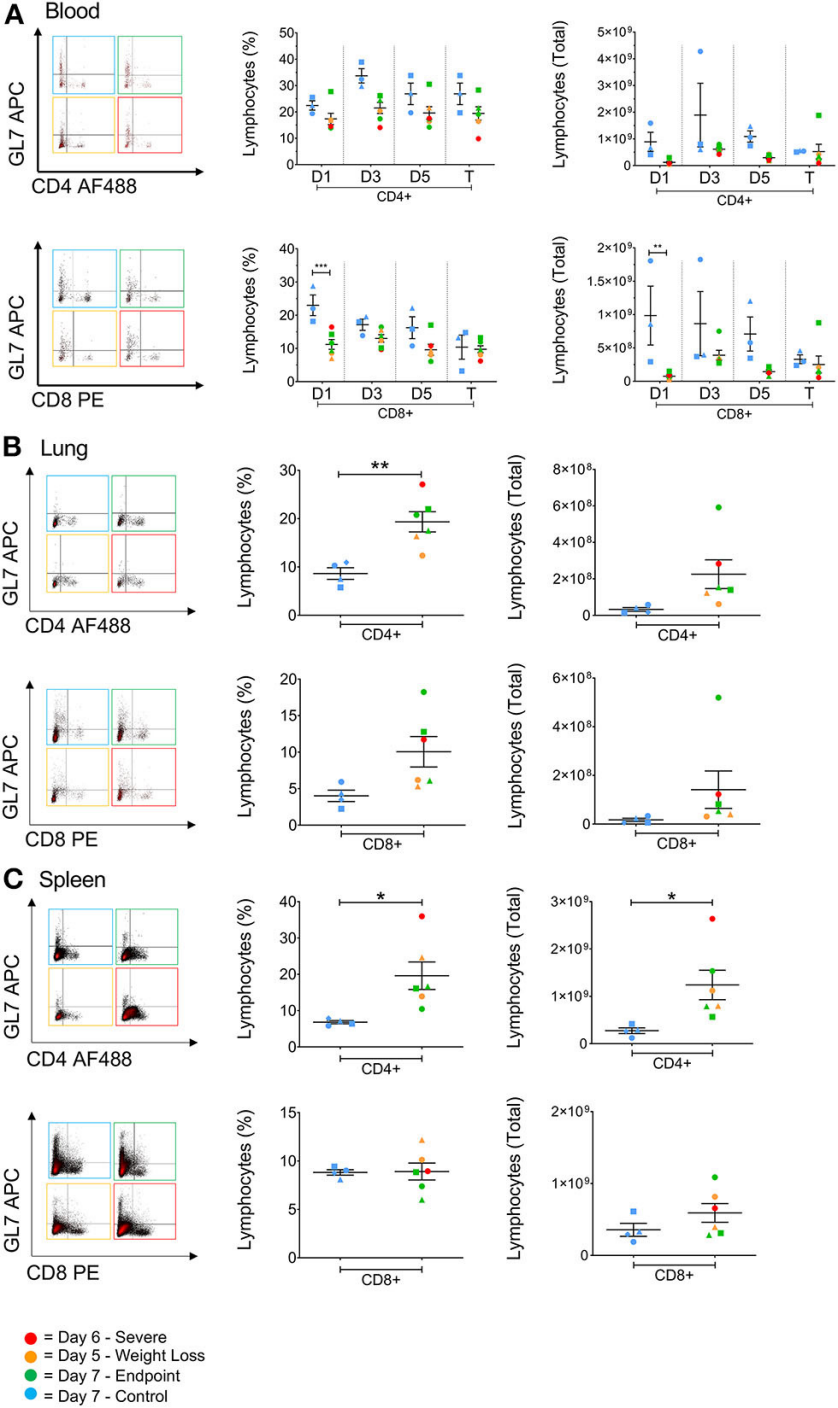
The effect of H7N9 on T lymphocyte subsets. Representative FACS plots of CD4^+^ and CD8^+^ T cell subsets plotted against GL7 activation marker, with percentage of positive cells and total cell numbers **(A)** in the blood over time, **(B)** in the lung, and **(C)** in the spleen. Time points marked with (*) indicate *p* < 0.05, (**) indicates *p* < 0.01, and (***) indicates *p* < 0.001. For blood samples, *n* = 3 for control, rather than *n* = 4, due to insufficient cell numbers recovered for one ferret.

Depletions in T cell numbers in the spleen and lungs have previously been reported for highly pathogenic avian strains H5N1 and H5N6 in both chickens and mice (6, 26). Similarly, patients that died from H7N9 infection were found to have lower T cell numbers in the blood compared to those that survived (5). Therefore, in order to assess lymphocyte involvement in disease progression we investigated the cellular responses associated with H7N9-infection both in the blood at several timepoints, and in the tissues at endpoint. Here, we found that in the blood the numbers of CD4^+^ and CD8^+^ T cells in infected ferrets were generally lower than the control ferrets, particularly for the CD8^+^ T cell population at day 1 (*p* < 0.001). Nevertheless, these T cell populations remained low but relatively stable over the 7 days of infection (Figure 5A). The GL7 monoclonal antibody can be used as a marker activated T lymphocytes. However, GL7 expression was absent on the T cell subsets examined. Interestingly we observed significantly higher numbers of CD4^+^ T cells in the spleen (*p* = 0.0272) and in the lungs (*p* = 0.0048) of infected ferrets, and a trend toward higher numbers of CD8^+^ T cells in the lungs (Figures 5B,C), indicating a T cell response to H7N9 infection at a key site of infection.

### Antigen Presenting Cell-Like Activation During H7N9 Infection in Tissues

**Figure 6:**
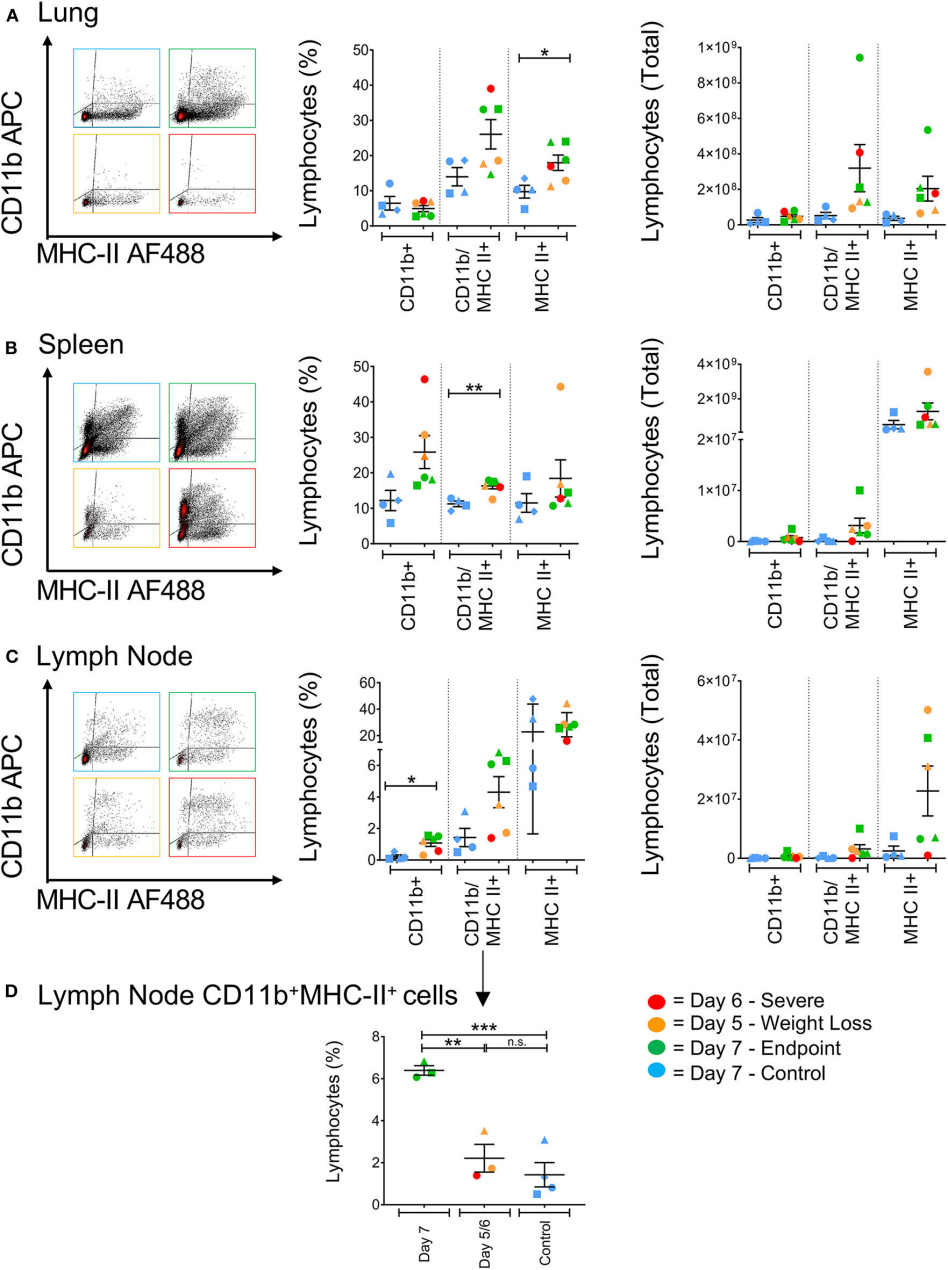
The effect of H7N9 infection on antigen presenting cell subsets. Representative FACS plots of CD11b^+^ and MHC-II^+^ antigen-presenting cell subsets, with percentage of positive cells and total cell numbers **(A)** in the lung, **(B)** in the spleen, and **(C)** in the mediastinal lymph node. **(D)** Ferrets had significantly greater levels of CD11b^+^MHC-II^+^ cells in the lymph node at Day 7 compared to earlier time points and the control animals. Data marked with (*) indicate *p* < 0.05, (**) indicates *p* < 0.01, and (***) indicates *p* < 0.001.

Antigen presenting cells (APCs), such as macrophages residing in the lungs, have previously been recognized as key pro-inflammatory responders during H5N1, H7N7, and H7N9 infections in mice (10). Cells expressing higher levels of CD11b^+^ have been shown to directly kill virally-infected cells in humans, and act as important antigen-presenting cells by upregulating MHC class II expression in the ferret model correlating to reduced pathology associated with influenza infection (17, 27). In general, H7N9-infected ferrets showed higher levels of CD11b/MHC-II dual-expressing cells (CD11b^+^MHC-II^+^) in the spleen, lung and lymph nodes compared to the control ferrets (Figures 6A–C). At the site of infection in the lungs, infected ferrets showed significantly higher levels of MHC-II single-positive cells (MHC-II^+^, *p* = 0.0286). Conversely, CD11b single-positive cells (CD11b^+^) from infected ferrets were found at elevated levels in the spleen and lymph nodes. Individually, we did observe that the ferrets euthanised on days 5 and 6 had higher CD11b^+^ cells in the spleen, suggesting a less activated and immature phenotype, and a trend toward lower CD11b^+^MHC-II^+^ cells in the lymph nodes when compared to the other ferrets. When analyzed by time point, there are statistically greater proportions of these cells in the ferrets that survived until trial endpoint compared to the controls (*p* = 0.0009) and the earlier timepoint ferrets (*p* = 0.0039, Figure 6D). As such, as these markers are often attributed to antigen-presenting cell maturation in the mouse model (28), APC activation in response to H7N9 infection may be associated with disease severity seen in the ferret model.

## Discussion

The recent SARS-CoV-2 outbreak has brought a sharp focus onto zoonotic viruses causing severe global pandemics. While sustained human-to-human transmission of avian-origin H7N9 viruses has yet to occur, if a zoonotic outbreak of these high-consequence viruses were to occur, the results could be a pandemic much like the SARS-CoV-2 outbreak but with a virus which has potential for much higher mortality rate. As such, avian influenza viruses continue to present as a major global health concern, with one of the key concerns being what makes these viruses cause such severe consequences in mammalian hosts.

Influenza-related symptoms of ferrets more closely resemble human clinical symptoms than that of mice (16, 29), with outbred ferret populations giving a more accurate representation of the genetic variability seen in human populations compared to clonally inbred mouse colonies. However, husbandry requirements and a general lack of reagents for ferrets limits the scope of experimental research using these animals to date. These issues are exacerbated by Biosafety Level 3 (BSL3) requirements for pathogens such as H7N9, which make immunological experiments in ferrets difficult to undertake. As a result, ferret cellular immunology is still a developing field of research in the context of influenza viruses, with only a handful of studies investigating changes in leukocyte populations following influenza virus infection (17–19). For avian-origin influenza viruses such as H7N9, little work has been done to investigate how cellular subsets are affected by viral infection, with the bulk of experiments using AI in the ferret model focusing on classifying viruses through clinical outcome-based pathogenesis and transmission studies (30–32). Thus, our study aimed to give new insight into how H7N9 affects mammalian hosts in the ferret model with a focus on cellular subsets.

The ferrets in our viral challenge presented clinical outcomes with different severities. Five of the six ferrets showed little to no clinical signs, nevertheless only three survived until study endpoint without complications. Two ferrets reached the ethical weight loss cut-off of ≥10% by day 5 post-infection, giving us two different time points to assess the immune response. Furthermore, one ferret showed a quite different disease outcome, in which the ferret presented an escalation of clinical signs and was euthanised for ethical reasons at day 6 post-infection. Other studies have previously shown clinical variation between strains of H7N9 (15), however most studies with A /Anhui/1/2013 (H7N9) show ferrets presenting only mild clinical signs (1, 4, 33). To our knowledge, this is the first reported result showing variable disease outcome in an animal study using a single low pathogenicity H7N9 strain, in which one ferret showed severe clinical outcomes. While the findings of this study are novel for this model, we were limited by the number of animals that could be used in this study and thus limited by our statistical power. Furthermore, there are limitations in directly comparing ferrets euthanised on different days as, particularly for our serological data, it is difficult to discern whether the results are a function of observed severity or differences in the sampling time. As such, we believe the results from our observational study can provide a rationale for developing future ferret studies with greater numbers. It is also worth noting that ferrets used for these experiments are outbred animals, which may suggest that other combinations of host factors may be contributing to the variability seen in clinical presentation.

The airway pathology in these infected ferrets was variable, with different degrees of severity observed in the six animals. These findings were generally consistent with pathology observed in other studies in which ferrets were infected with LPAI H7N9 or pdm09 (H1N1) viruses (4, 31, 32, 34–36). While the day 6 ferret which showed worsened disease progression did exhibit the most severe lung pathology, the lack of viral antigen in and around these lesions (Supplementary Figure 1) means they cannot conclusively be classified as being caused by the influenza infection, and thus we are hesitant to classify disease presentation in this ferret as like a high pathogenicity infection based on the histopathology findings regardless of the increase in clinical signs.

Hypercytokinemia has frequently been associated with worsened disease progression in cases of severe AI infections, therefore we aimed to assess the induction of pro-inflammatory cytokines in this ferret model of infection. Upregulation of pro-inflammatory cytokines such as TNF-α and IL-6 commonly observed following H7N9 infection in both cell culture (14, 37) and in severe human cases, which follow similar patterns of pathogenesis to infections with HPAI H5N1 viruses (5, 38, 39). Whilst the localized pro-inflammatory cytokine response to influenza infection has been characterized for circulating influenza strains in ferrets (40), examination into these responses for avian influenza viruses remains limited. Our study found limited induction of cytokine responses at the site of infection in the lungs and in lymphoid tissues, and while attenuated IFN responses have been previously reported in human bronchial epithelial cells infected with H7N9 (14), and in ferrets severely infected with seasonal influenza strains (41), the overall lack of cytokine induction was an unexpected finding. A limitation of this study was that these tissues could only be sampled at study endpoint, which may suggest the pro-inflammatory response had abated at the site of infection by the time of sampling given that these responses typically occur in short timeframes of 24–48 h post-infection (42), though a lack of an evident cytokine response in the day 6 ferret still presenting clinical signs and shedding live virus was surprising. Furthermore, levels of IFN-γ were below the threshold of detection by ELISA in the serum of infected ferrets, and a previous study has shown IFN-γ detectable in the lungs of seasonal influenza-infected ferrets from day 5 post-infection at low levels, with greater detection observed from days 8–11 (19). These results suggest both timing and sampling may be critical for future studies to best capture the overall IFN-γ response to H7N9, if there is such a response occurring. Sampling in the blood of infected ferrets over the course of the study also found no large-scale upregulation of the cytokines tested, with only the early innate cytokine MCP1 showing an average fold increase >5-fold compared to the controls at day 1 post-infection. However, in the day 6 ferret, decreases in both IFN-γ (25-fold) and IL-6 (24-fold) where observed at day 5 post-infection, coinciding with an escalation in clinical signs in this animal. The decrease in IL-6 in particular was unexpected, as previous studies have implicated higher levels of IL-6 correlating to worsened disease progression (41). Our findings here suggest an immune dysregulation, rather than an over-activation, for worsened disease progression, and may be attributed to other factors in the cellular response.

Immunological work to determine the innate and adaptive responses to these viruses has mostly been conducted from infected patients or in the mouse model. Human cases are often marked by leukopenia, though these measurements are routinely made via hematology rather than flow cytometric analysis (43, 44). In other species however, we have previously described the lymphopenia for highly pathogenic strains of avian influenza, measuring splenic CD8^+^ T cells following infection with highly pathogenic H5N6 in chickens via FACS analysis (6). In this study, CD8^+^ T cells levels were relatively stable. A loss of lymphocytes detected via hematological analysis at day 1 (data not shown) was reconciled with our FACS data showing a significant difference between CD8^+^ T cells in the blood, in which control animals had much higher levels compared to the infected animals. However, this loss was short-lived, as no further significant differences were observed across the study time points in the blood. This data is suggestive of a potential early “transient lymphopenia” of circulating leukocytes, which has previously been identified in one study during influenza infection in ferrets, and is also a phenomenon seen in humans following influenza A infection (17, 45). Our data appears to show CD8^+^ T cells levels in the blood of infected animals remaining relatively and consistently low across time points, whilst greater variation was observed in the uninfected control levels.

Furthermore, reagents for FACS analysis in the ferret are still relatively novel, often not well-characterized, and heavily reliant on cross-reactivity from other species. While CD4^+^ and CD8^+^ T cells have previously been examined in influenza-infected ferrets (18), little work has been conducted for innate cell subsets. Myeloid-derived and antigen-presenting cells (APCs) have only been identified and analyzed in a handful of ferret studies, with one such study speculating that CD11b^+^ cells are likely granulocytes such as neutrophils (17, 18). As such, we have used these studies, together with mouse and human studies (46–49), as a basis for possible classification of APCs, with CD11b^+^MHC-II^+^ cells suggested as likely “mature” or “activated” APCs. However, further work is needed to classify these innate cell subsets in the ferret model.

APCs such as alveolar macrophages have been recognized as key early responders against influenza virus infection, as they mount robust pro-inflammatory cytokine responses within 24 h of infection (27). While our study found no significant differences in APC numbers in infected lungs compared to control numbers, there was a trend toward much greater levels of MHC-II^+^ APCs in the lung, especially in our cell counts. Rather, it was in the peripheral tissues that we found variability in the APC subsets. CD11b^+^MHC-II^+^ “mature” cells were found in the spleens of infected ferrets at significantly higher levels (*p* = 0.0029), however the trend toward higher CD11b^+^ cells in the earlier time point ferrets suggests a higher level of immature myeloid cells, or the presence of pro-inflammatory granulocytes; however, the latter is less likely due given the lack of pro-inflammatory cytokines detected in the spleen. Moreover, in the draining lymph node a significant increase in CD11b^+^ cells (*p* = 0.0153) was observed. Interestingly, in the lymph node the 3 day seven ferrets showed noticeably higher levels of CD11b^+^MHC-II^+^ “activated” cells, with statistically greater proportions of these cells compared to the controls (*p* = 0.0009) and the earlier timepoint ferrets (*p* = 0.0039), which may suggest that these APC responses do not occur as early as day 5.

This study found increased pathology in the ferret model diverges from the commonly observed pathogenesis markers such as lymphopenia and hypercytokinemia, suggesting another varied pathway that H7N9 viruses can cause severe disease in mammalian hosts. Further investigation into the ferret model may allow for better characterization of these outcomes and assist in increasing our knowledge of these viruses in preparation for any potential pandemic events.

## Data Availability Statement

All datasets generated for this study are included in the article/Supplementary Material.

## Ethics Statement

The animal study was reviewed and approved by Australian Animal Health Laboratories (AAHL) Animal Ethics Committee, CSIRO.

## Author Contributions

All authors agreed on the final draft of the manuscript prior to submission. WH wrote the first draft and revised the manuscript. TN, KK, JBu, SS, JBi, JP, AB, and DL reviewed and edited the manuscript.

## Conflict of Interest

The authors declare that the research was conducted in the absence of any commercial or financial relationships that could be construed as a potential conflict of interest.
