# Supplementary figures and images for: The Dynamics of the Ferret Immune Response During H7N9 Influenza Virus Infection
Source: Front Immunol. 2020 Sep 24;11:559113. doi: 10.3389/fimmu.2020.559113 (PMC7541917; doi:10.3389/fimmu.2020.559113)

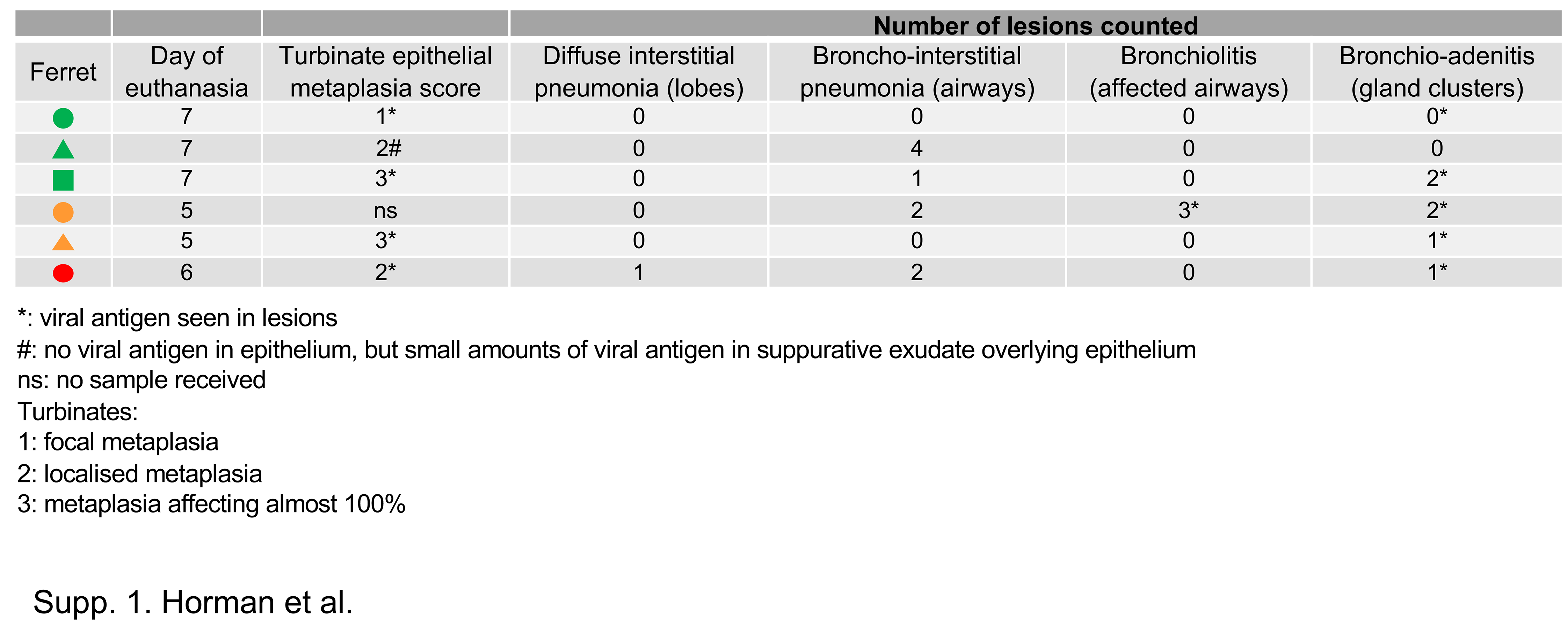

Supplement: Supplementary file 1 [file Image_1.TIF]
